# Supplementary material for: Five years of pharmaceutical industry funding of patient organisations in Sweden: Cross-sectional study of companies, patient organisations and drugs
Source: PLoS One. 2020 Jun 24;15(6):e0235021. doi: 10.1371/journal.pone.0235021 (PMC7313941; doi:10.1371/journal.pone.0235021)
Supplement: S1 Table — (DOCX) [file pone.0235021.s001.docx]

**Supplementary Table 1.** **Coding manual for payment categories and goals**

| **Payment category**  *Payment categories relate to the overall form of the payment. They were established in Ozieranski et al [1] on the basis of the codes used by the European Federation of Pharmaceutical Industries and Associations (EFPIA) to categorise pharmaceutical company payments to healthcare organisations (e.g. hospitals, universities, medical associations), specifically, “grants”, “contributions to costs of events”, “travel, accommodation and registration fees”, “fees for service and consultancy”, and “sponsorship”. These payment categories were supplemented with an inductive approach for categories unique to patient organisation payment descriptions in the UK [1]. Key words alongside iterative readings of payment descriptions were used when applying the codes.* | | |
| --- | --- | --- |
| **Code** | **Definition** | **Examples (UK data; see [1, 2])** |
| Contributions to costs of events organised by recipients or third parties | Payments relating to costs of events organised by the recipient patient organisation or a third party | “Payment for stand - hospitality suite + set up at WFH Meeting 8-12 July 2012”  “Exhibitor fees for stand at NI Diabetes foot conference”  “Sponsorship of the Pharmacy Management National Seminar “Transforming Your Pharmaceutical Care” for Northern Ireland”. |
| Fees for service and consultancy (including travel and accommodation) | Payments made in exchange for a patient organisation’s services | “Contracted service for Expert Consulting Fees for various projects (Financial support)”  “We paid Genetic Alliance UK to write an article on rare disease for the Pfizer Life website”  “Consultancy fee and travel expenses for representative to attend patient asthma advisory board meeting in Paris” |
| Form of funding unclear | Unclear overall form of funding | “Asthma UK Burden of Asthma project”  “Patient / public education and  awareness on disease”  “Safer Sun Campaign” |
| Grants | Payments described as grants which do not fall into any of the other payment categories | “Diabetes family support group grant towards books, table clothes, banners”  “Funding to engage with the rheumatology community in Northern Ireland to raise awareness of Ankylosing Spondylitis and the setting up of a NASS branch network to encourage affordable regular access to physiotherapy.”  “Grant to support the development of HoFH educational materials” |
| More than one distinct payment form | More than one payment form described in the payment description, making it impossible to determine the ultimate form. | “Event support; Organisation support; Patient education”  “We donated colleague volunteering time by arranging and providing a 'Step Up' challenge day to assist Meningitis Now in solving a business challenge. We provided a venue and a light lunch on the day.”  “Supporting the Patient Association to manage their Freedom of Information Request on C. difficile management to all hospital trusts. Also included Astellas Corporate Membership to The Patients Association.” |
| Partnership arrangements | Payments relating to partnership and collaborative arrangements and activities | “We entered into a partnership working agreement to co-develop research about the experiences and expectations of older people and cancer. Age UK and Pfizer commissioned Picker Institute Europe to undertake the fieldwork. The fieldwork costs were funded by Pfizer.”  “To support the Corporate Partnership Programme.”  “Set up and running of a –sing with us” choir for Surrey area. 3 year collaboration.” |
| Sponsorship of participation at events organised by drug companies | Payments made towards patient organisations’ participation in events hosted by drug companies | “Four delegate spaces on Pfizer thinkspace, a series of free development workshops open to the UK patient organisation community (estimated value £1,800)”  “NRAS facilitated for a patient to attend a workshop at an internal Chugai Pharma UK meeting to talk about the impact of rheumatoid arthritis”  “Shire Patient Association Development Workshop.” |
| Sponsorships | Payments specified as sponsorship (other than sponsorship of participation at events organised by drug companies) of a patient organisation’s activities | “Sponsorship of Liver Wellness Programme”  “Co‐sponsorship of Haematology Educations Consortium for 2014.”  “Sponsorship: Lung Cancer Answering your Questions pack (Financial support)” |
| Support and help | Payments specified as providing support for a patient organisation’s activities | - “Support to help with the ongoing activity and general administration”  “To support the cost of translation and development of patient information booklets, factsheets and a drug chart.”  “Support towards the Online and interactive side  effect checker allowing patients to id and rank side  effects they are experiencing project” |
| Travel, accommodation and registration fees | Payments for travel, accommodation, and attendance expenses incurred by patient organisations when attending events | “MSD covered the travel expenses for a patient speaker  at MSD's internal Immunology Team Training Day, which took place in Q4 2011. MSD made the payment to NACC, who in turn reimbursed the patient speaker's travel costs.”  “Travel expenses for launch meeting of an Alcohol Policy report at the European Commission”  “Grant to support the costs incurred by the Patient Group members during a congress attendance or event (registration fee, meals, flights and  accommodation).” |
| **Payment goal**  *Payment goal was coded based on the codes established in Ozieranski et al [1] and close iterative reading of payment descriptions. The overall principle for coding of goals was to capture the specific nature of funded activities as much as possible. When coding payment goals we looked for the main purpose of activities funded by drug companies.* | | |
| **Code** | **Definition** | **Examples (UK data; see [1, 2])** |
| Accessing or paying for organisation's expertise | Payments made in exchange for a patient organisation’s expertise services and expert knowledge | “Internal training to Chugai Pharma UK on the impact of rheumatoid arthritis on the patient & family, August 2012”  “For the group's consultative services.”  “Honoraria payment to Rarer Cancers Foundation for Speaker at PACE Advisory Board (Financial support).” |
| Advocacy, campaigning, and disease awareness | Contributions towards activities relating to patient advocacy, campaigning, and disease awareness | “£5,000 paid to support the Compare Your Care Campaign to raise awareness of standards of asthma care and self‐management support in the NHS around the UK.”  “Educational Literature The contribution is provided to produce materials for the national infertility awareness campaign.”  “Funding for Advocacy Support Officer” |
| Communication-in general, media, meetings, online, publications, skills development | Payments related to all types of communications activities | “Support and sponsor of pelvic floor pads, including print and logo placement within the document. Copies are available for the sponsor to distribute, in addition to the Bladder and Bowel Foundation.”  “Sanofi provided funding for stand space at a scientific meeting organised by Parkinson's UK - Movement Disorders Disease Specific Advisory Group Launch/Study Day on Wednesday 4th May 2016. (BCUHB event)”  “Development of the Action for Pulmonary Fibrosis website and of developing a package of patient- facing leaflets.” |
| Education and training | Payments related to education and training activities including study days, workshops, and educational leaflets for patients | “For the provision of four study days for midwives on the subject of pre eclampsia during 2013”  “Two delegate spaces on Pfizer thinkspace, a series of free development workshops open to the UK patient organisation community (estimated value £900).”  “UCB supported the costs of printing 6,000 flyers that were used to promote the organization's new e- learning modules that were created for HCPs” |
| Funding for awards | Payments supporting patient organisations’ award events | “Brook Clinical Leads Conference Brook/FPA Sexual Health Awards”  “Donation to support the UK Sexual Health Awards 2015 helping to recognise and reward best practice in sexual health. Bayer HealthCare is proud sponsor of the Rosemary Goodchild Award, celebrating excellence in sexual health journalism.”  “Sponsorship of MS Society Awards.” |
| Inputting to organisation's work via membership, partnership, sponsorship or support | Core payments supporting the general running of the recipient patient organisation, including corporate membership | “Asthma UK Corporate Gold membership”  “We paid for annual membership of the organisation.”  “Sanofi provided corporate partnership to the Juvenile Diabetes Research Centre.” |
| More than one distinct purpose mentioned | More than one payment goal in a given payment description | “Provision of support for COBF to continue their helpline and work in raising awareness of overactive bladder”  “Grant given to a Patient Group for fundraising events, communication, advocacy and to cover maintenance and infrastructure related costs (e.g. Building refurbishing, start up costs, equipment,  membership, etc).”  “Grant to support the Buddy Awards, administration, nominations process, awards ceremony, media coverage” |
| Patient support | Payments related to the facilitation of activities focused on patient support | “Lung Cancer Patient Support Groups ‐ Facilitator pack development”  “We made a donation to support the Rarer Cancers Foundation’s provision of services to patients and carers affected by rarer cancers, which includes the provision of a telephone helpline and the online Rarer Cancers Forum.”  “We made a charitable donation to support a series of patient and family events to support people living with lung cancer.” |
| Policy engagement | Payments for policy related activities | “Policy and Campaigning support work for 2012”  “Policy report on alcohol harm reduction”  “Sponsorship of Dementia policy lunch (closed) events, co- hosted between Alzheimer's Society and MSD at the Labour, Conservative and Liberal Democrat party conferences.” |
| Research | Payments for research related activities | “Research grant to support the collection of epidemiological  data on visual impairment”  “We provided a grant as part of a joint working collaboration to provide an information day for prospective research grant applicants applying to the Health Services Research Call.”  “To carry out survey of their patient membership with Short Bowel Syndrome (SBS) on Home Parenteral Nutrition (HPN). To capture data on a number of issues related to burden of illness, impact of quality of life on patient and family/carers.” |
| Support for fundraising | Payments contributing towards the fundraising efforts of patient organisations | “We made two donations to add to a colleague donation as part of our matched funding programme.”  “Contribution towards fundraising event”  “We made a charitable donation to add to a colleague donation as part of our matched funding programme.” |

**References:**

1. Ozieranski P, Rickard E, Mulinari, S. Exposing drug industry funding of UK patient organisations. BMJ. 2019;365:l1806.

2. Rickard E, Ozieranski P, Mulinari S. Evaluating the transparency of pharmaceutical company disclosure of payments to patient organisations in the UK. Health Policy. 2019;123(12):1244- 50.
