# Supplementary material for: Five years of pharmaceutical industry funding of patient organisations in Sweden: Cross-sectional study of companies, patient organisations and drugs
Source: PLoS One. 2020 Jun 24;15(6):e0235021. doi: 10.1371/journal.pone.0235021 (PMC7313941; doi:10.1371/journal.pone.0235021)
Supplement: S2 Table — (DOCX) [file pone.0235021.s002.docx]

**Supplementary Table 2.** Drug industry funders of Swedish patient organisations (2014-18)

| Company | Value of payments. € (%)^1^ | n (%)^2^ |
| --- | --- | --- |
| Pfizer | 954 234 (14.8) | 92 (6.9) |
| AbbVie | 731 902 (11.3) | 146 (10.9) |
| Sanofi | 447 812 (6.9) | 87 (6.5) |
| Roche | 404 049 (6.3) | 131 (9.8) |
| Novartis | 403 508 (6.3) | 95 (7.1) |
| GlaxoSmithKline | 383 346 (5.9) | 43 (3.2) |
| Janssen | 335 870 (5.2) | 85 (6.4) |
| Bayer | 260 900 (4.0) | 57 (4.3) |
| Celgene | 243 880 (3.8) | 64 (4.8) |
| Amgen | 214 103 (3.3) | 64 (4.8) |
| Boehringer Ingelheim | 208 146 (3.2) | 30 (2.2) |
| Astellas | 201 272 (3.1) | 48 (3.6) |
| Eli Lilly | 182 681 (2.8) | 44 (3.3) |
| Takeda | 170 908 (2.6) | 31 (2.3) |
| AstraZeneca | 155 792 (2.4) | 33 (2.5) |
| CSL Behring | 129 730 (2.0) | 22 (1.6) |
| Biogen | 126 091 (2.0) | 21 (1.6) |
| Shire | 121 149 (1.9) | 41 (3.1) |
| Merck Sharp & Dohme | 85 299 (1.3) | 22 (1.6) |
| Merck | 81 587 (1.3) | 25 (1.9) |
| Bristol-Myers Squibb | 79 549 (1.2) | 28 (2.1) |
| Novo Nordisk | 68 610 (1.1) | 32 (2.4) |
| Gilead | 62 632 (1.0) | 7 (0.5) |
| Otsuka | 50 571 (0.8) | 8 (0.6) |
| Swedish Orphan Biovitrum | 49 444 (0.8) | 5 (0.4) |
| UCB | 41 858 (0.6) | 6 (0.4) |
| Ipsen | 36 935 (0.6) | 17 (1.3) |
| Teva | 35 124 (0.5) | 2 (0.1) |
| Tillotts | 34 778 (0.5) | 6 (0.4) |
| Baxter | 22 868 (0.3) | 8 (0.6) |
| Orion Pharma | 22 111 (0.3) | 3 (0.2) |
| LEO Pharma | 17 317 (0.3) | 5 (0.4) |
| Lundbeck | 14 248 (0.2) | 4 (0.3) |
| Genzyme | 14 247 (0.2) | 2 (0.1) |
| Actelion | 12 773 (0.2) | 5 (0.4) |
| Santen Pharma | 10 959 (0.2) | 1 (0.1) |
| Octapharma | 6 756 (0.1) | 4 (0.3) |
| Nigaard | 3 705 (0.1) | 2 (0.1) |
| Mundipharma | 3 361 (0.1) | 1 (0.1) |
| Viropharma | 3 021 (0.1) | 1 (0.1) |
| Medivir | 2 879 (0.1) | 1 (0.1) |
| AGA | 2 240 (0.1) | 1 (0.1) |
| Chiesi | 2 216 (0.1) | 2 (0.1) |
| Nordic Drugs | 1 462 (0.1) | 1 (0.1) |
| Almirall Aps | 975 (0.1) | 1 (0.1) |
| Sandoz | 329 (0.1) | 1 (0.1) |
| Total | 6 449 224 (100) | 1 337 (100) |

^1^ Percent of total value of payments (€6,449,224)

^2^ Percent of total number of payments (1,337)
