# Supplementary material for: Five years of pharmaceutical industry funding of patient organisations in Sweden: Cross-sectional study of companies, patient organisations and drugs
Source: PLoS One. 2020 Jun 24;15(6):e0235021. doi: 10.1371/journal.pone.0235021 (PMC7313941; doi:10.1371/journal.pone.0235021)
Supplement: S3 Table — (DOCX) [file pone.0235021.s003.docx]

**Supplementary Table 3.** Top 10 drug industry payments to Swedish patient organisations (2014-18)

| **Company** | **Patient organisation (translated)** | **Value of payment € (year of funding)** | **Payment description (abbreviated)** |
| --- | --- | --- | --- |
| **Pfizer** | Swedish Breast Cancer Association | 353 179 (2016) | Support for creation of website for disseminating information on breast cancer |
| **Pfizer** | Swedish Breast Cancer Association | 97 497 (2018) | Further support of website for disseminating information on breast cancer |
| **Boehringer Ingelheim** | Swedish Diabetes Association | 83 555 (2018) | Support for developing report on Type 2 Diabetes |
| **Eli Lilly** | Swedish Diabetes Fund | 69 097 (2014) | Support for event to increase knowledge of childhood diabetes and raise awareness of World Diabetes Day and the need for financial support to research on diabetes in children and adolescents. |
| **Takeda** | Association for Gastrointestinal diseases | 61 918 (2017) | Support for development and evaluation of digital patient tool |
| **Janssen** | Association for Gastrointestinal diseases | 61 534 (2016) | Support for a communication project about Crohn’s disease |
| **AbbVie** | Association for Gastrointestinal diseases | 61 904 (2015) | Support for workshops targeting patients and health care; and for developing a patient support tool to increase knowledge on Inflammatory Bowel Disease |
| **Pfizer** | National Association for Young Rheumatics | 56 378 (2017) | Support of a member survey on how it is to live with rheumatic disease. |
| **AbbVie** | Young with psoriasis | 49 170 (2016) | Support of an information project to increase knowledge about psoriasis and to support those living with Psoriasis/Psoriatic Arthritis |
| **Sanofi** | FH-Sweden | 48 391 (2015) | Financial support to raise awareness of Familial Hypercholesterolemia (FH) |
